# Supplementary material for: Evaluation of Temozolomide and Fingolimod Treatments in Glioblastoma Preclinical Models
Source: Cancers (Basel). 2023 Sep 8;15(18):4478. doi: 10.3390/cancers15184478 (PMC10527257; doi:10.3390/cancers15184478)
Supplement: Supplementary file 1 [file cancers-15-04478-s001.zip › cancers-2561895-supplementary.pdf]

cancers

MDPI

Article

Evaluation of Temozolomide and Fingolimod Treatments in Glioblastoma Preclinical Models

Mélodie Davy †, Laurie Genest †, Christophe Legrand, Océane Pelouin, Guillaume Froget, Vincent Castagné and Tristan Rupp *

Porsolt SAS, ZA de Glatigné, 53940 Le Genest-Saint-Isle, France; medavy@porsolt.com (M.D.); lgenest@porsolt.com (L.G.); tof.legrand@yahoo.fr (C.L.); opelouin@porsolt.com (O.P.); gfroget@porsolt.com (G.F.); vincent.castagne53@sfr.fr (V.C.)
* Correspondence: trupp@porsolt.com or rupptristan@hotmail.fr; Tel.: +33-(0)2-43-69-36-07
† These authors contributed equally to this work.

Supplementary Figure legends

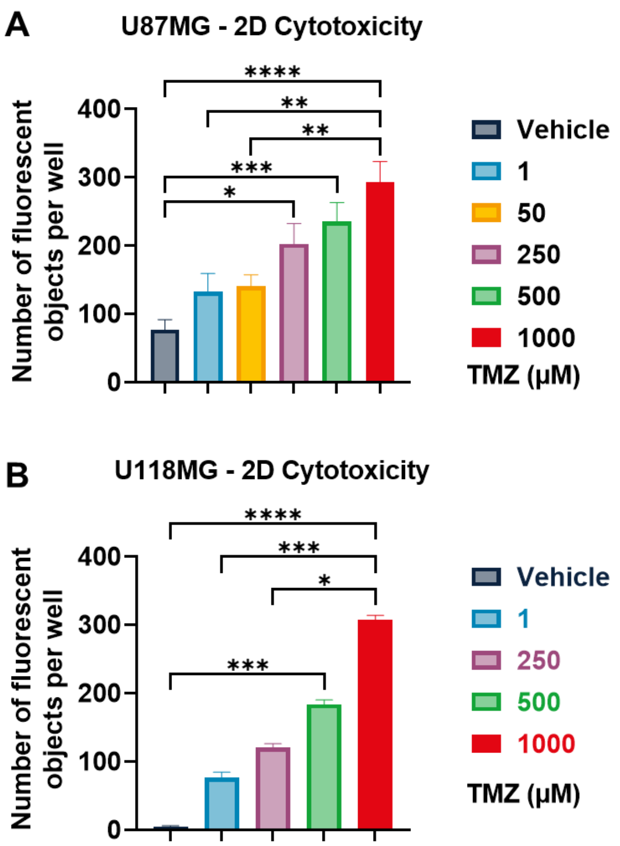

Figure S1. Effects of Temozolomide (TMZ) treatment on glioblastoma in 2D cell cytotoxicity assay. A-B. Dose-response effect of TMZ treatments on U87MG (A) and U118MG (B) cytotoxicity using image-based analysis. The number of fluorescent-positive dead cell was quantified per well. The assay was performed with triplicates from three (B) to six (A) independent experiments. Data represent mean and SEM. Statistical differences between the groups were determined using Kruskal-Wallis' test followed by Dunn's multiple comparisons test (* $p \leq 0.05$, ** $p \leq 0.01$, *** $p \leq 0.001$, **** $p \leq 0.0001$).

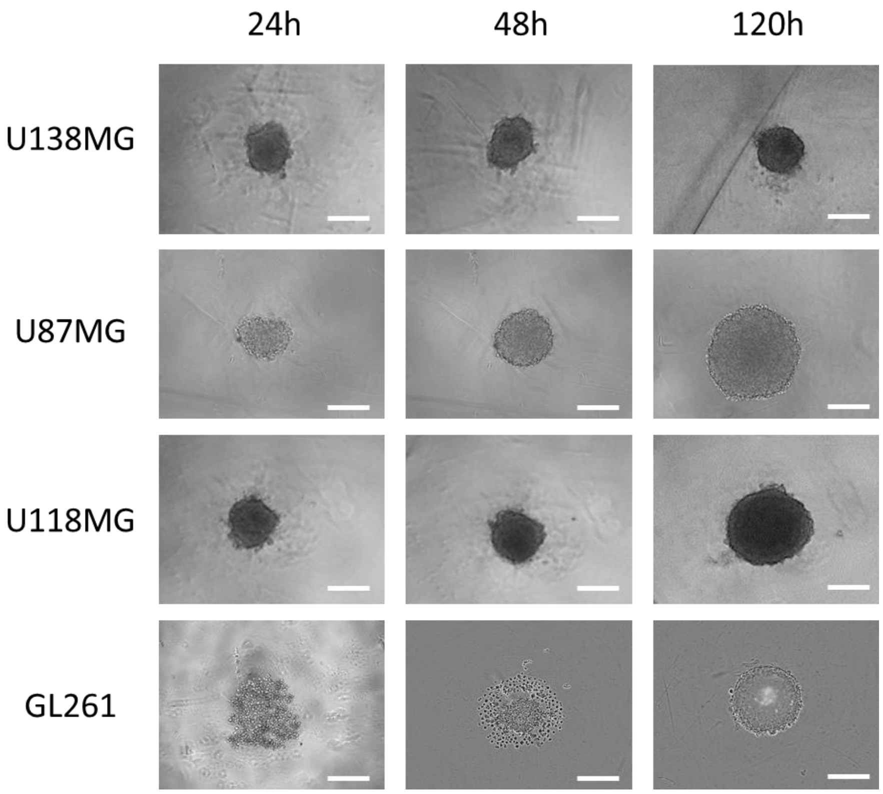

Figure S2. Evaluation of spontaneous spheroid formation using U138MG, U87MG, U118MG, and GL261 cells. Representative pictures of U138MG, U87MG, U118MG, and GL261 cells after 24, 48, and 120 hours of culture in 96 well plate CellCarrier Spheroid ULA Microplates™ (Perkin Elmer®) in culture medium. Scale = 300 µm.

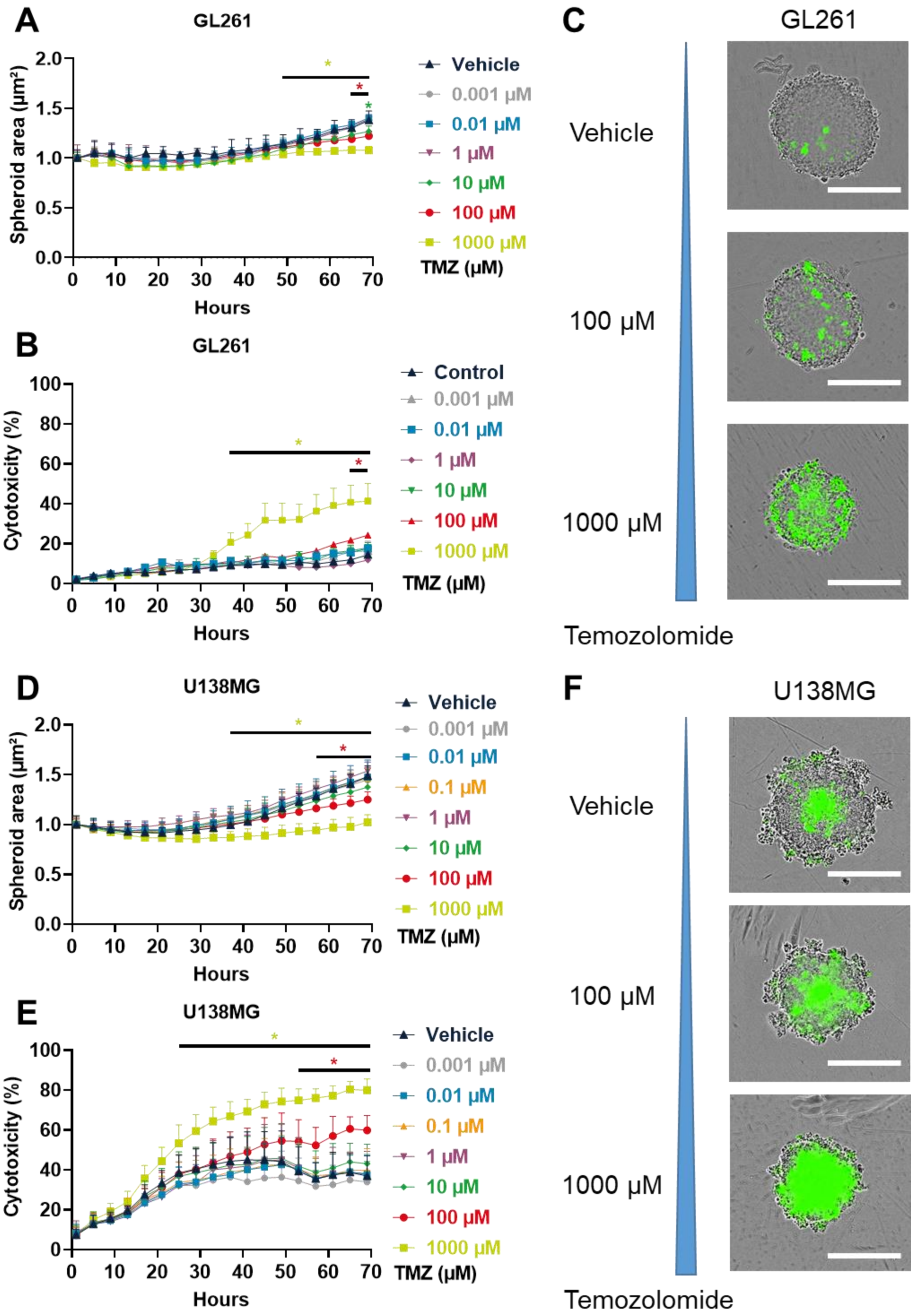

Figure S3. Effects of Temozolomide (TMZ) on glioblastoma cells in 3D tumor spheroid assay. A-F. Dose-response effect of TMZ treatment on GL261 (A-C) and U138MG (D-F) spheroid size (A and D) and cytotoxicity (B and E). The spheroid area and fluorescent-positive surface was quantified per spheroid (C and F). Representative pictures of cells after 69 hours' post-treatment with 0, 100, and 1000 µM of TMZ were shown. Scale = 500 µm. The assay was performed with 3 to 4 replicates from 2 to 3 independent experiments. Data represent mean and SEM. Statistical differences were determined using a mixed-effects model (REML, groups and time as factor) and Bonferroni's multiple comparisons test (vs. control, * $p \leq 0.05$).

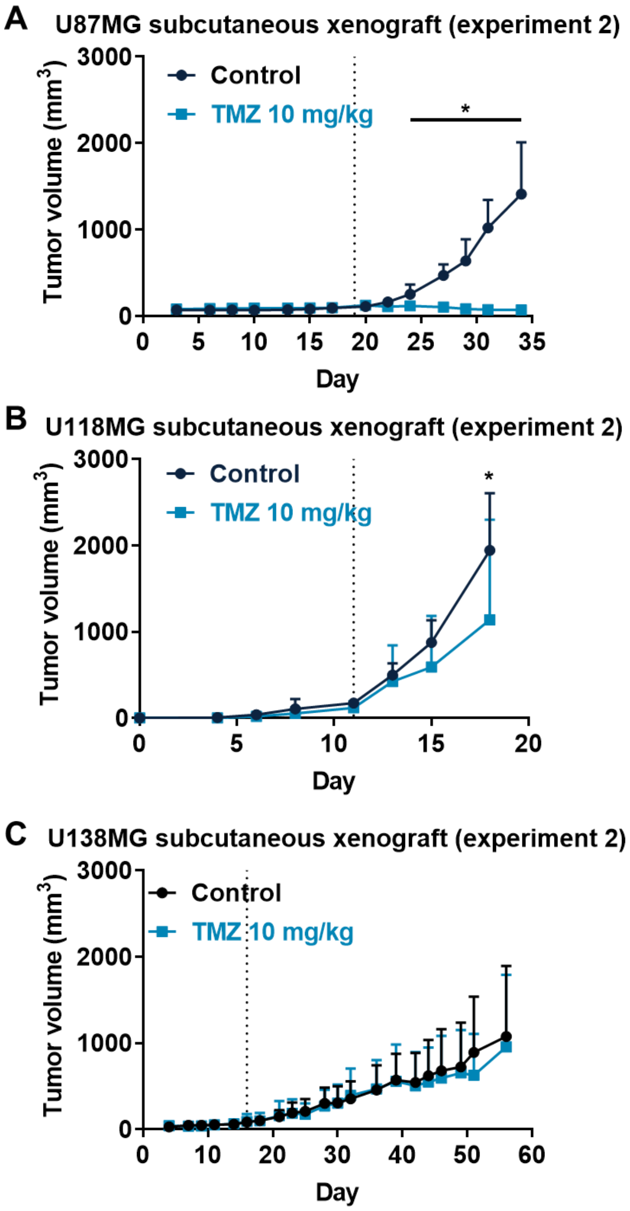

Figure S4. Effects of Temozolomide (TMZ) treatment in glioblastoma mouse subcutaneous xenograft models. A-C Impact of TMZ at 10 mg/kg administered 5 times a week (p.o.) on tumor volume in U87MG xenograft (A), U118MG xenograft (B), and U138MG xenograft (C) models. Discontinuous line highlights treatment beginning. Statistical differences between the groups were determined using a mixed-effects model (REML, groups and time as factor) followed by Bonferroni's multiple comparisons test (* $p \leq 0.05$). Data represent mean and SD. n=5-6 (A), n=6 (B), n=6 (C) mice per group at the start of treatments.

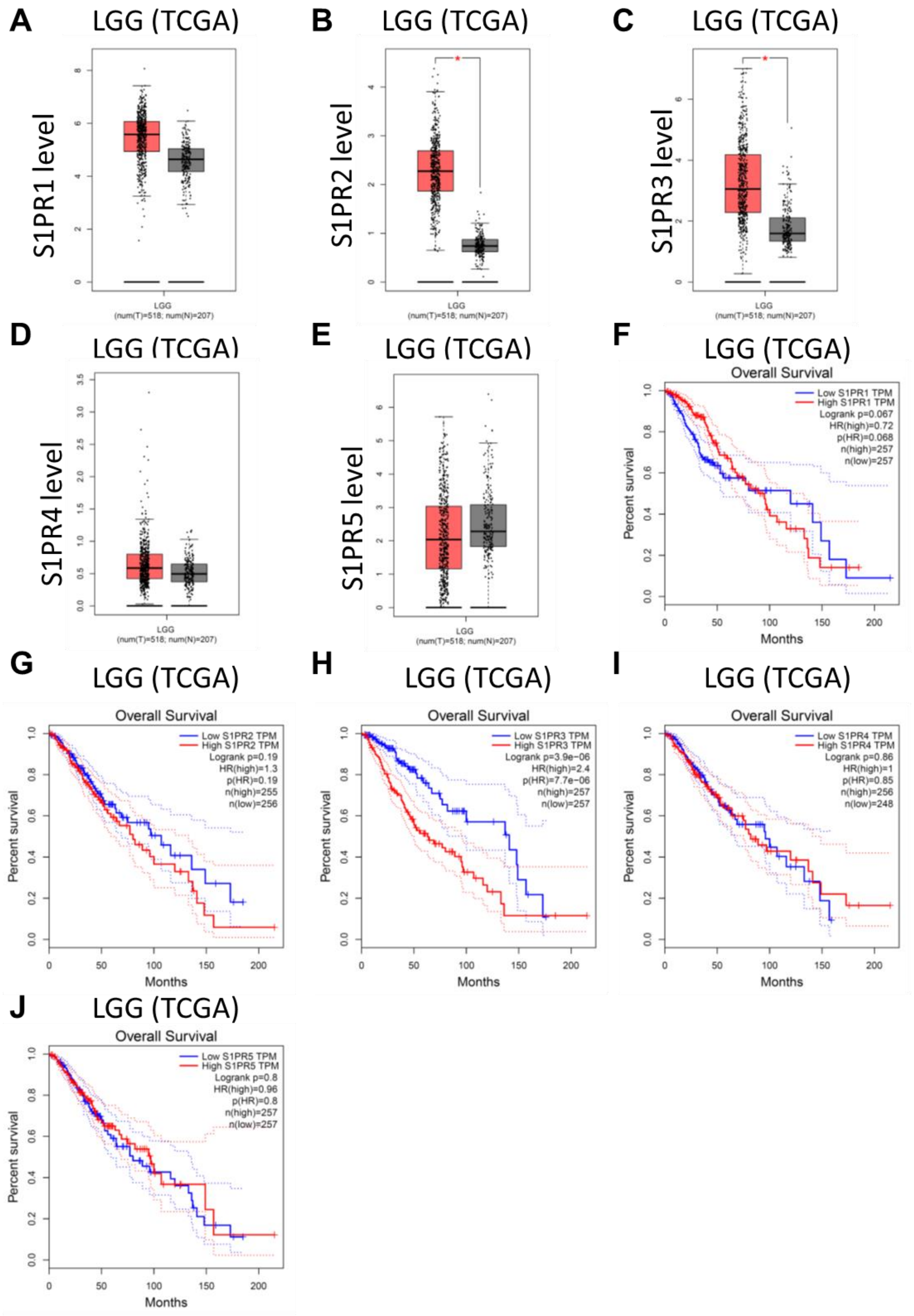

Figure S5. Analysis of publicly available clinical data of S1PR1, S1PR2, S1PR3, S1PR4, and S1PR5 expression in LGG patients from TCGA cohort. A-E S1PR2 and S1PR3 are significantly overexpressed in GBM tumors as compared to non-tumoral brains. S1PR1, S1PR4, and SP1R5 are not significantly affect despite an observable increase for S1PR1 and S1PR4. The number of patients in each group is indicated below the graphs (T=Tumor in red; N=normal non-tumoral brain in grey), p-values indicate the significance of expression difference between the groups of individuals by ANOVA. F-J. High expression of S1PR3 is correlated with poor overall survival (OS) of LGG patients. Expression of S1PR1, S1PR2, S1PR4, and S1PR5 are not correlated with OS of LGG patients. Patients were stratified in low or high-expressing groups according to the expression of the different genes using the median value. The number of patients in each group is indicated below the

graphs, p-values indicate the significance of survival difference between the groups of individuals by log-rank test.

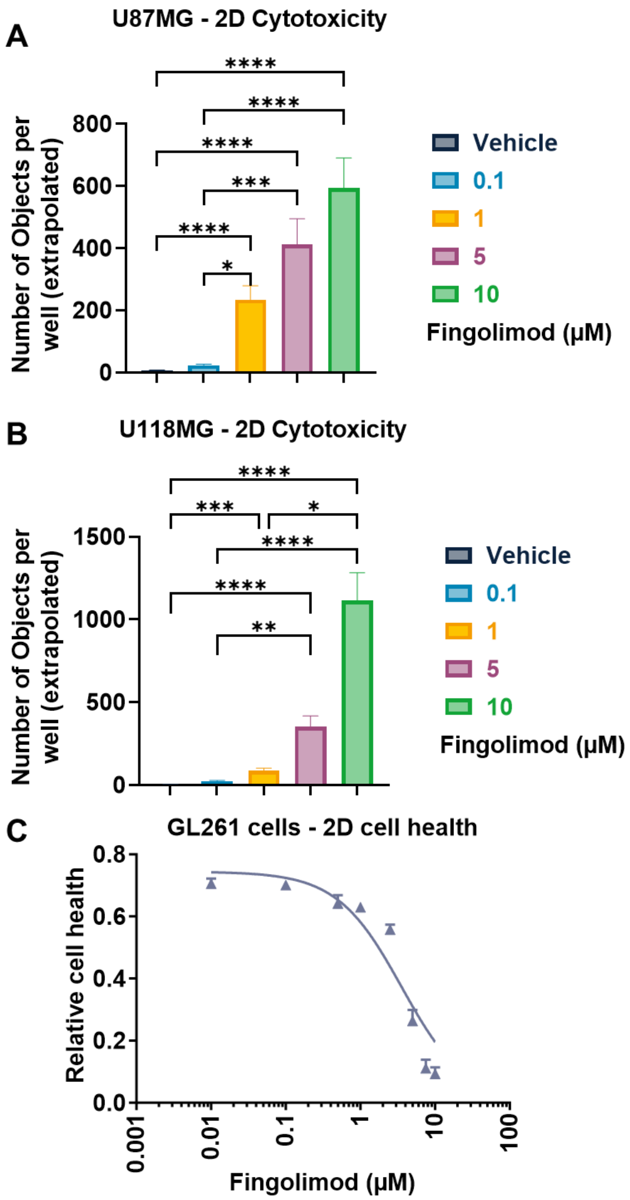

Figure S6. Effects of Fingolimod treatment on GBM in 2D cell cytotoxicity assay. A-B. Dose-response effect of Fingolimod treatment on U87MG (A) and U118MG (B) cytotoxicity using image-based analysis. The number of fluorescent-positive dead cell was quantified per well. The assay was performed with triplicates from three (B) to six (A) independent experiments. Data represent mean and SEM. Statistical differences between the groups were determined using Kruskal-Wallis' test followed by Dunn's multiple comparisons test (* $p \le 0.05$, ** $p \le 0.01$, *** $p \le 0.001$, **** $p \le 0.0001$). C. Dose-response effect of Fingolimod treatment on GL261 cell health using MTS-incorporation assay. Data represent mean and SEM.

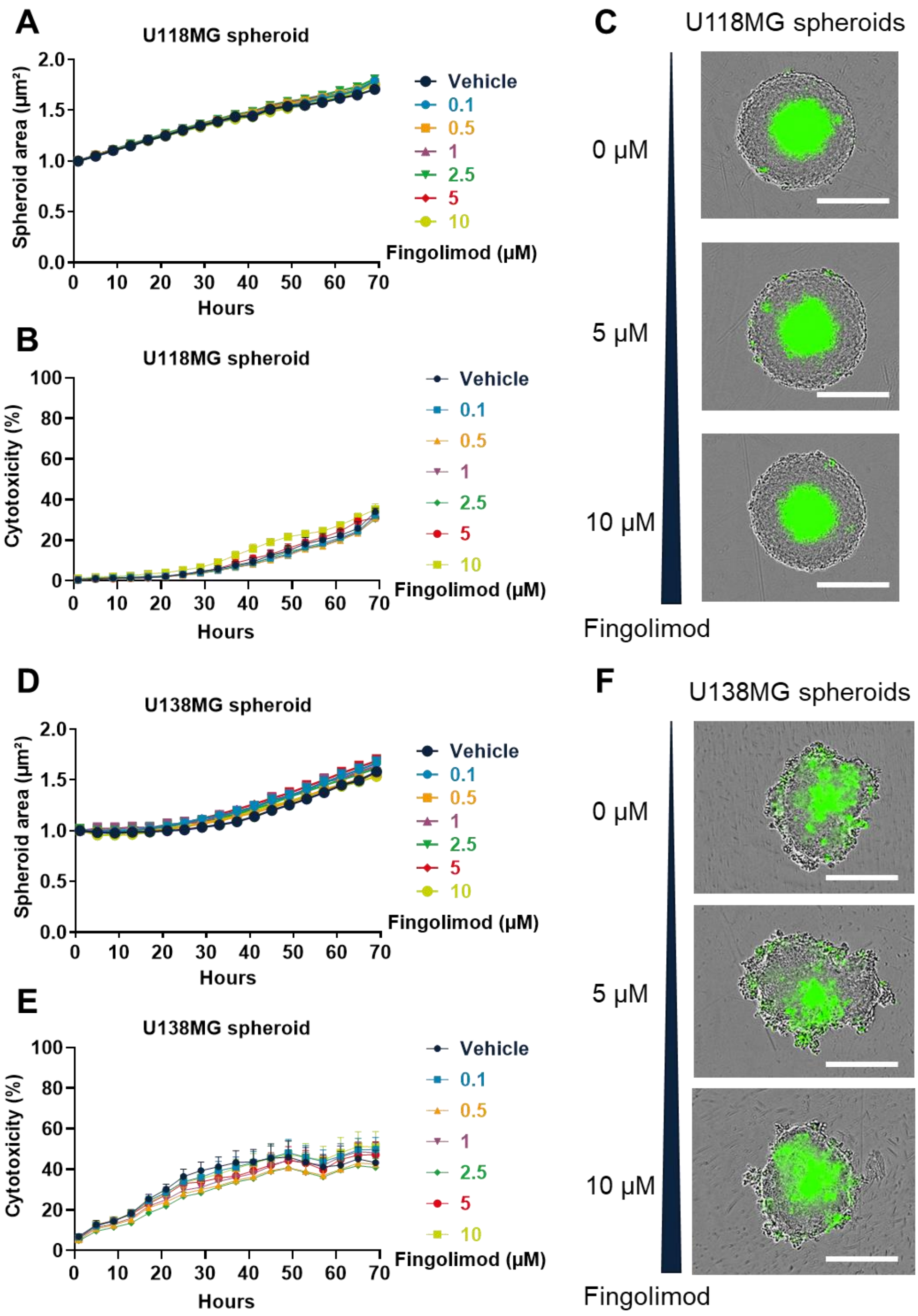

Figure S7. Effects of Temozolomide (TMZ) on glioblastoma cells in 3D tumor spheroid assay. A-F. Dose-response effect of TMZ treatment on U118MG (A-C) and U138MG (D-F) spheroid size (A and D) and cytotoxicity (B and E). The spheroid area and fluorescent-positive surface was quantified per spheroid (C and F). Representative pictures of cells after 69 hours' post-treatment with 0, 100, and 1000 µM of TMZ were shown. Scale = 500 µm. The assay was performed with 3 to 4 replicates from 2 to 3 independent experiments. Data represent mean and SEM. Statistical differences were determined using a mixed-effects model (REML, groups and time as factor) and Bonferroni's multiple comparisons test (vs. control, * $p \leq 0.05$).
